# Supplementary figures and images for: Entamoeba histolytica Develops Resistance to Complement Deposition and Lysis after Acquisition of Human Complement-Regulatory Proteins through Trogocytosis
Source: mBio. 2022 Mar 1;13(2):e03163-21. doi: 10.1128/mbio.03163-21 (PMC8941920; doi:10.1128/mbio.03163-21)

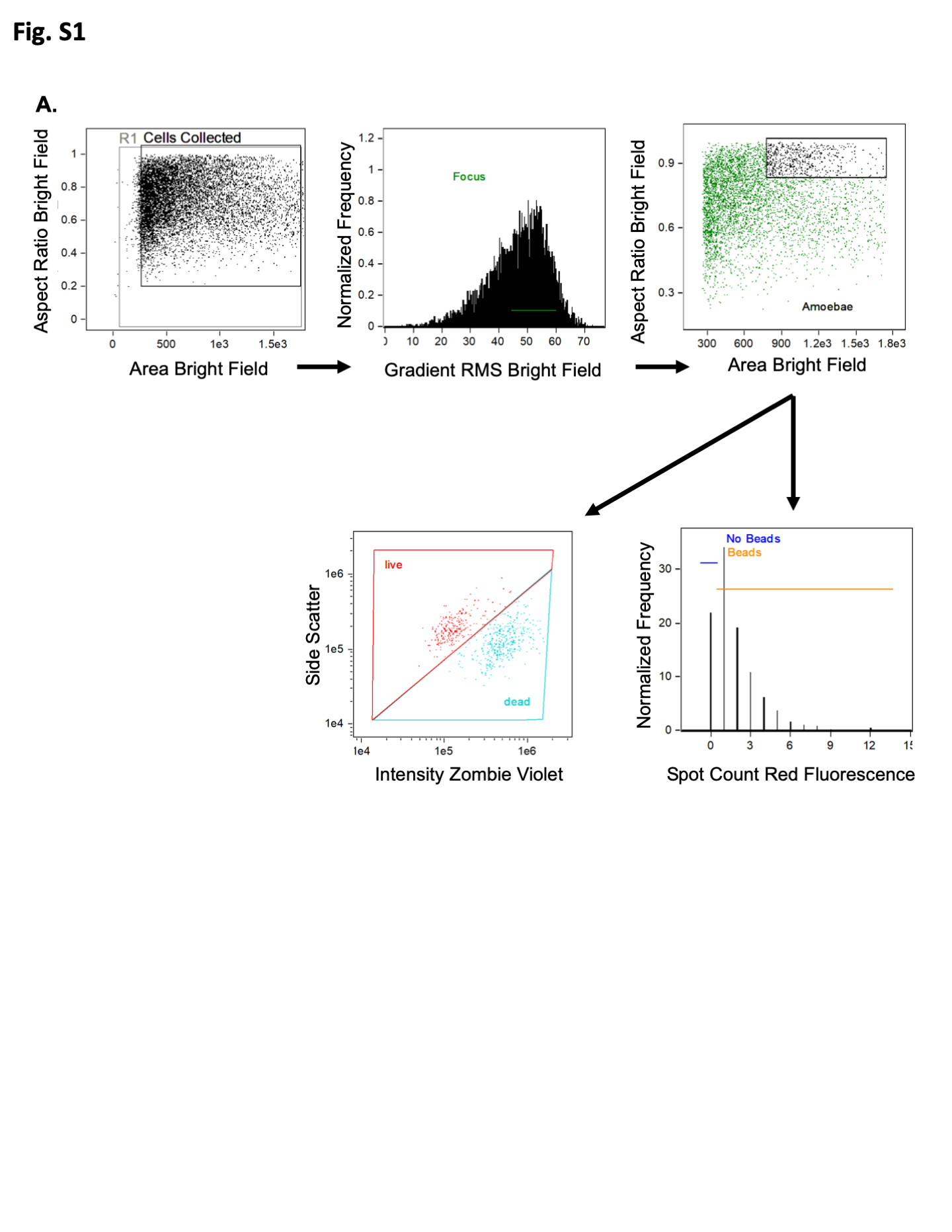

Supplement: FIG S1 [file mbio.03163-21-sf001.tif]

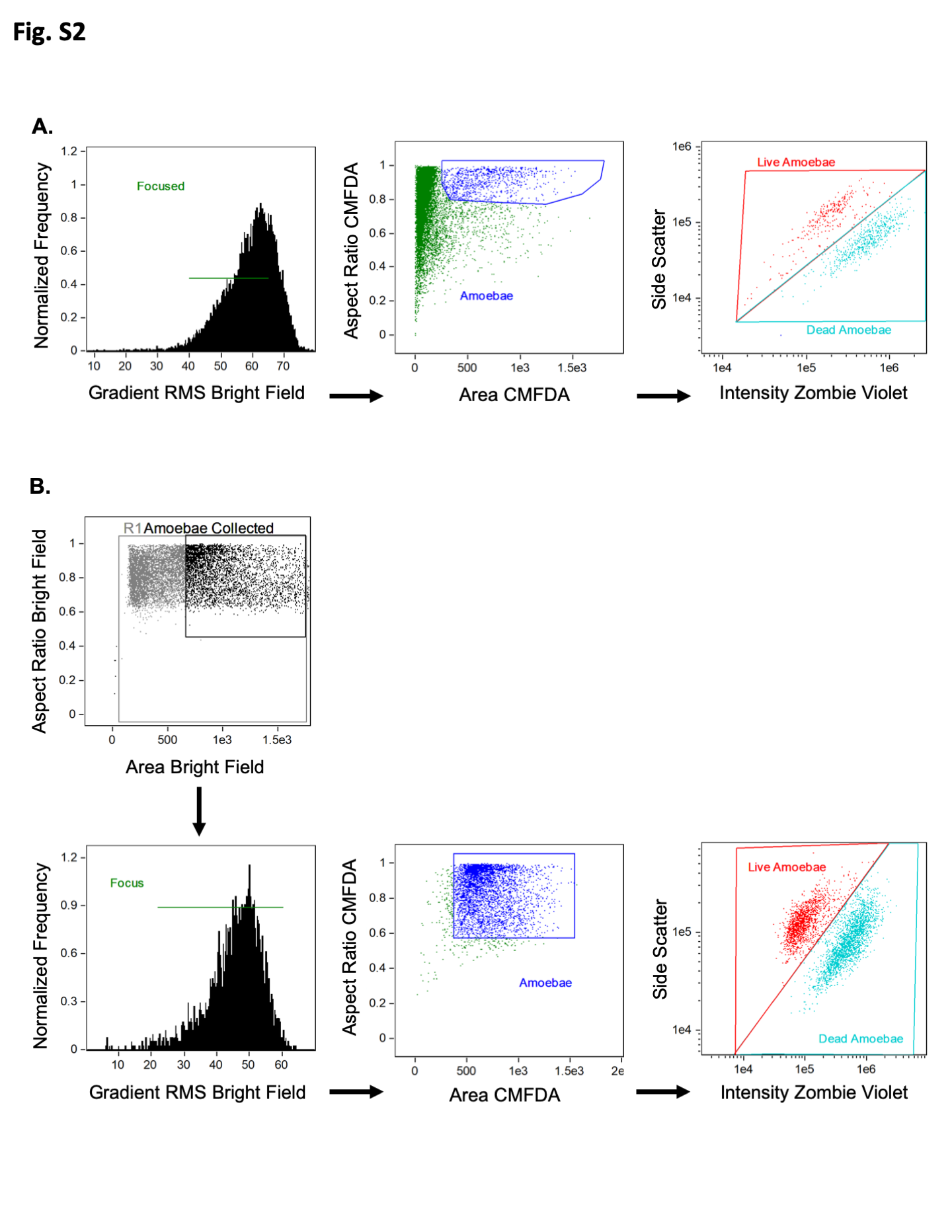

Supplement: FIG S2 [file mbio.03163-21-sf002.tif]

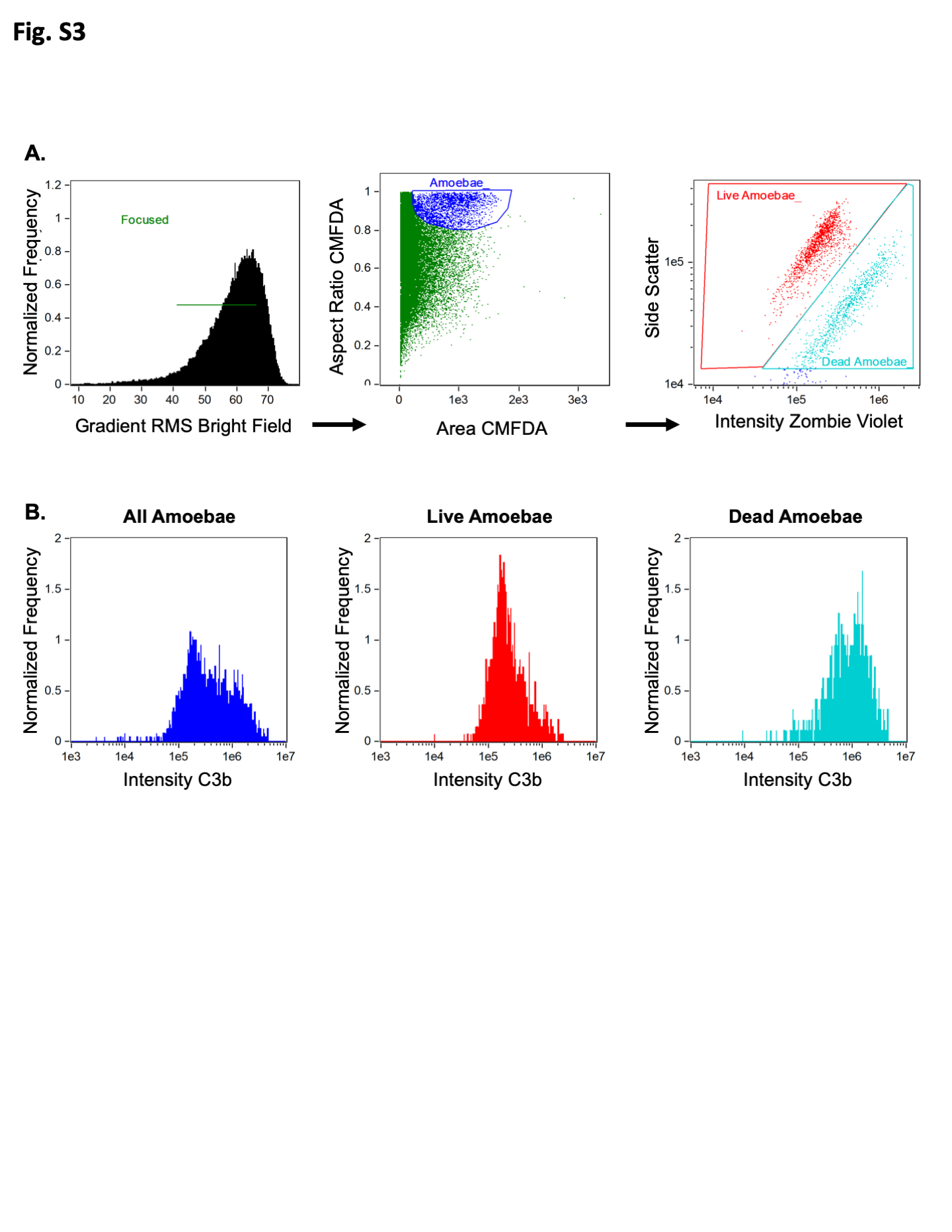

Supplement: FIG S3 [file mbio.03163-21-sf003.tif]

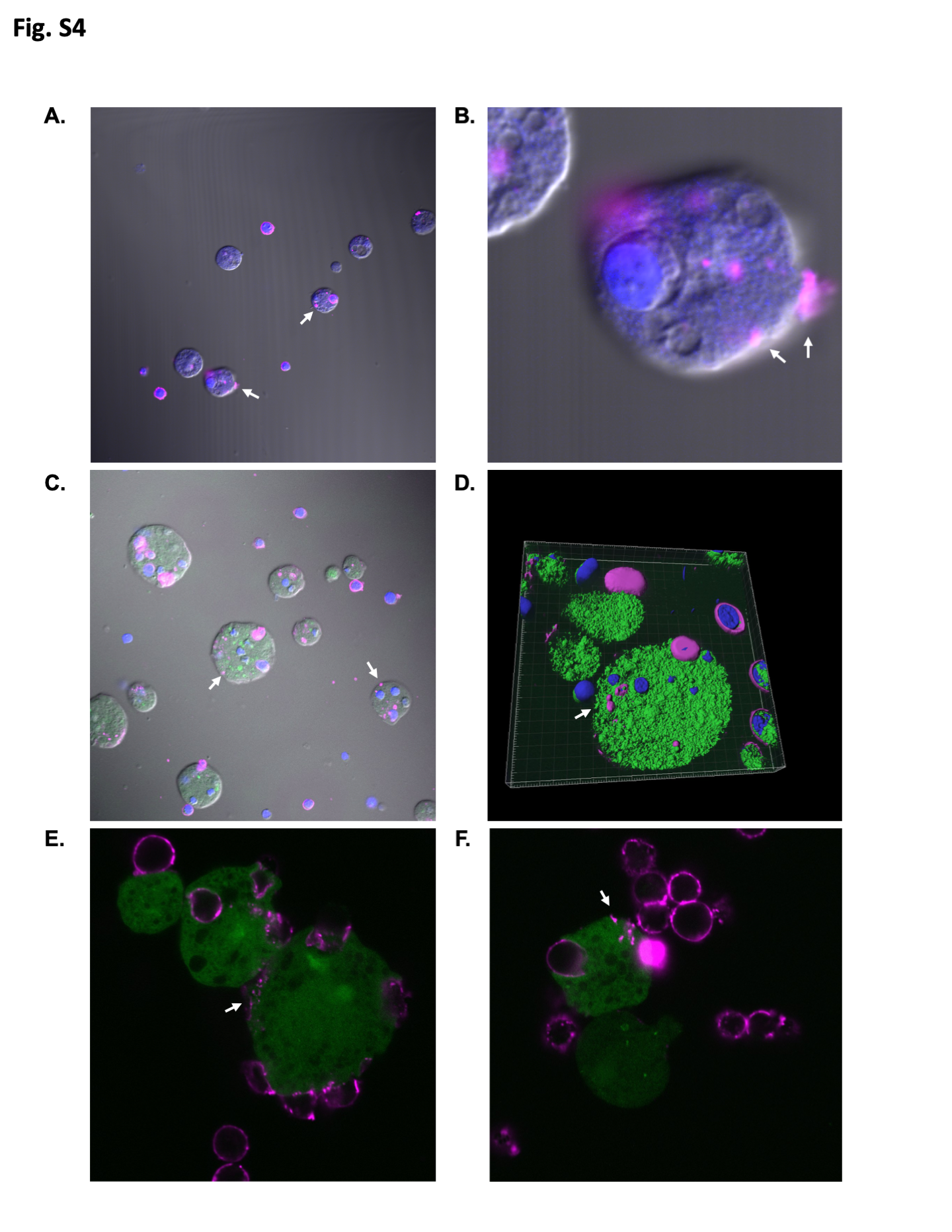

Supplement: FIG S4 [file mbio.03163-21-sf004.tif]

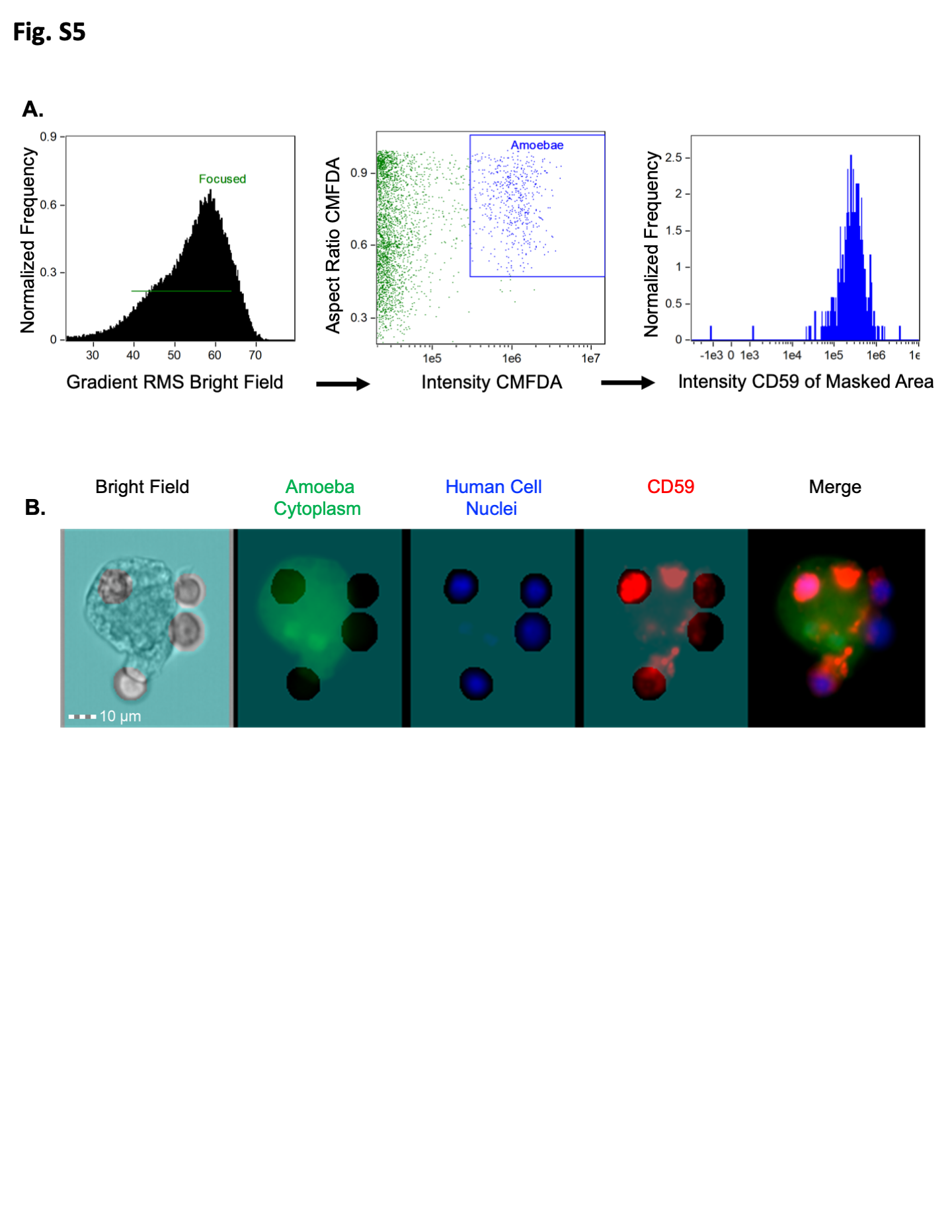

Supplement: FIG S5 [file mbio.03163-21-sf005.tif]

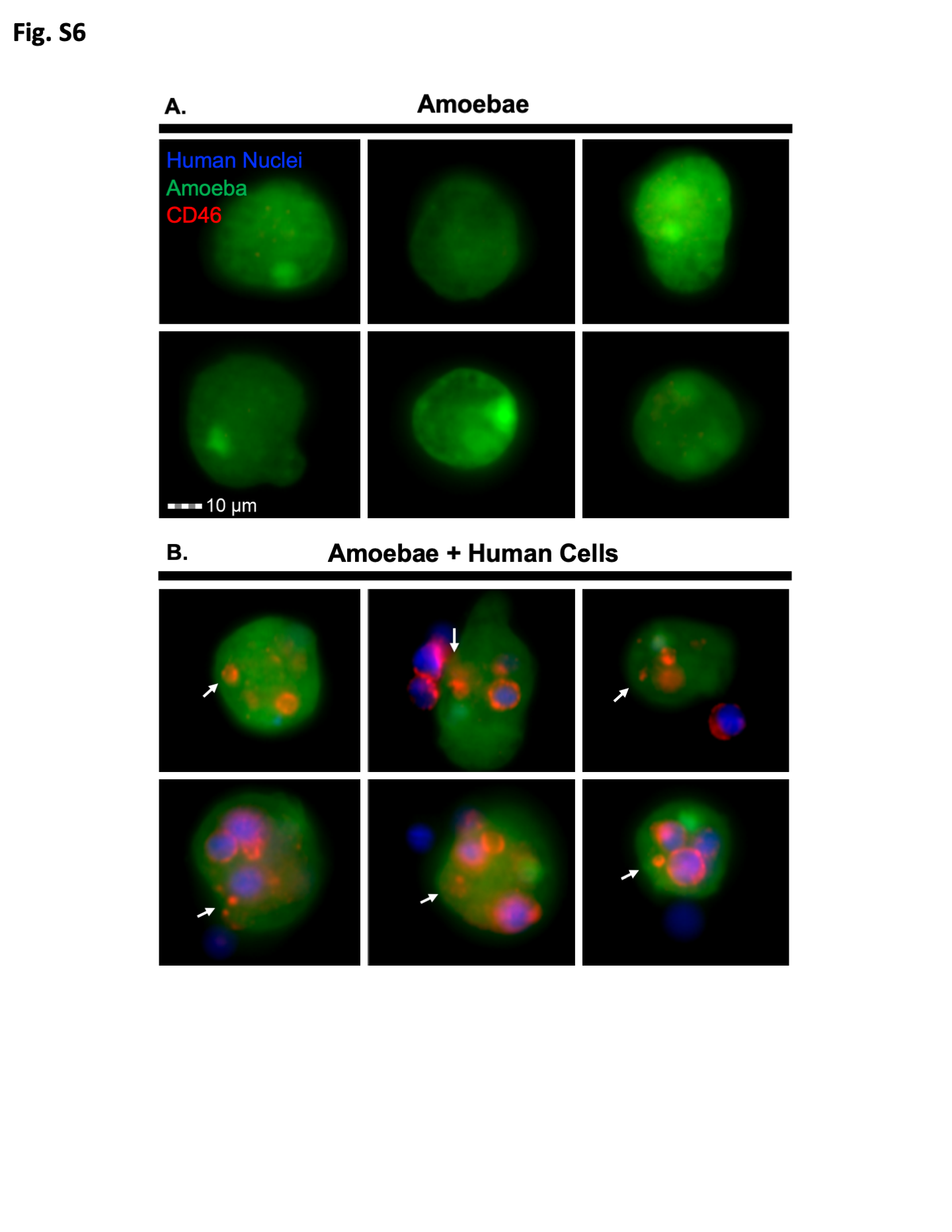

Supplement: FIG S6 [file mbio.03163-21-sf006.tif]

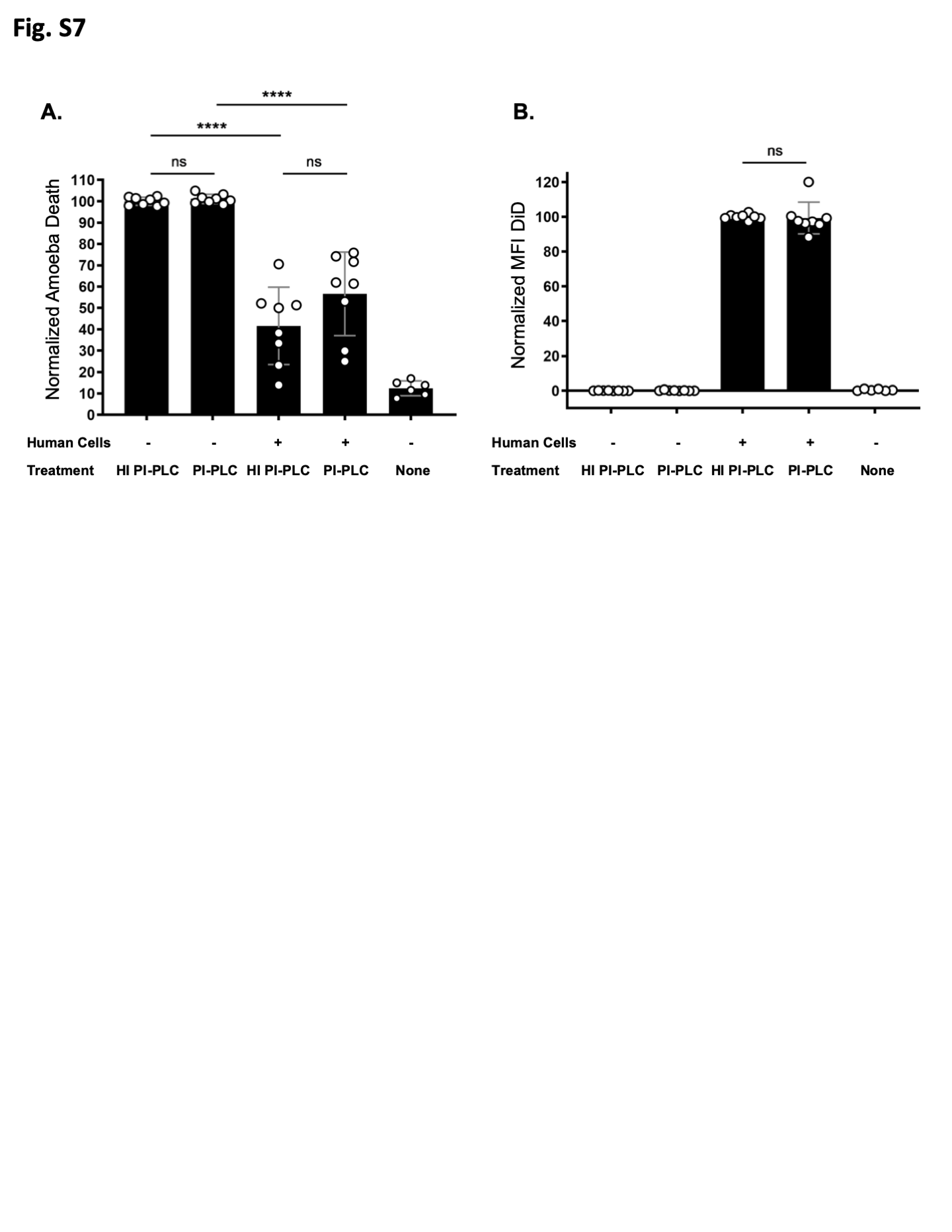

Supplement: FIG S7 [file mbio.03163-21-sf007.tif]

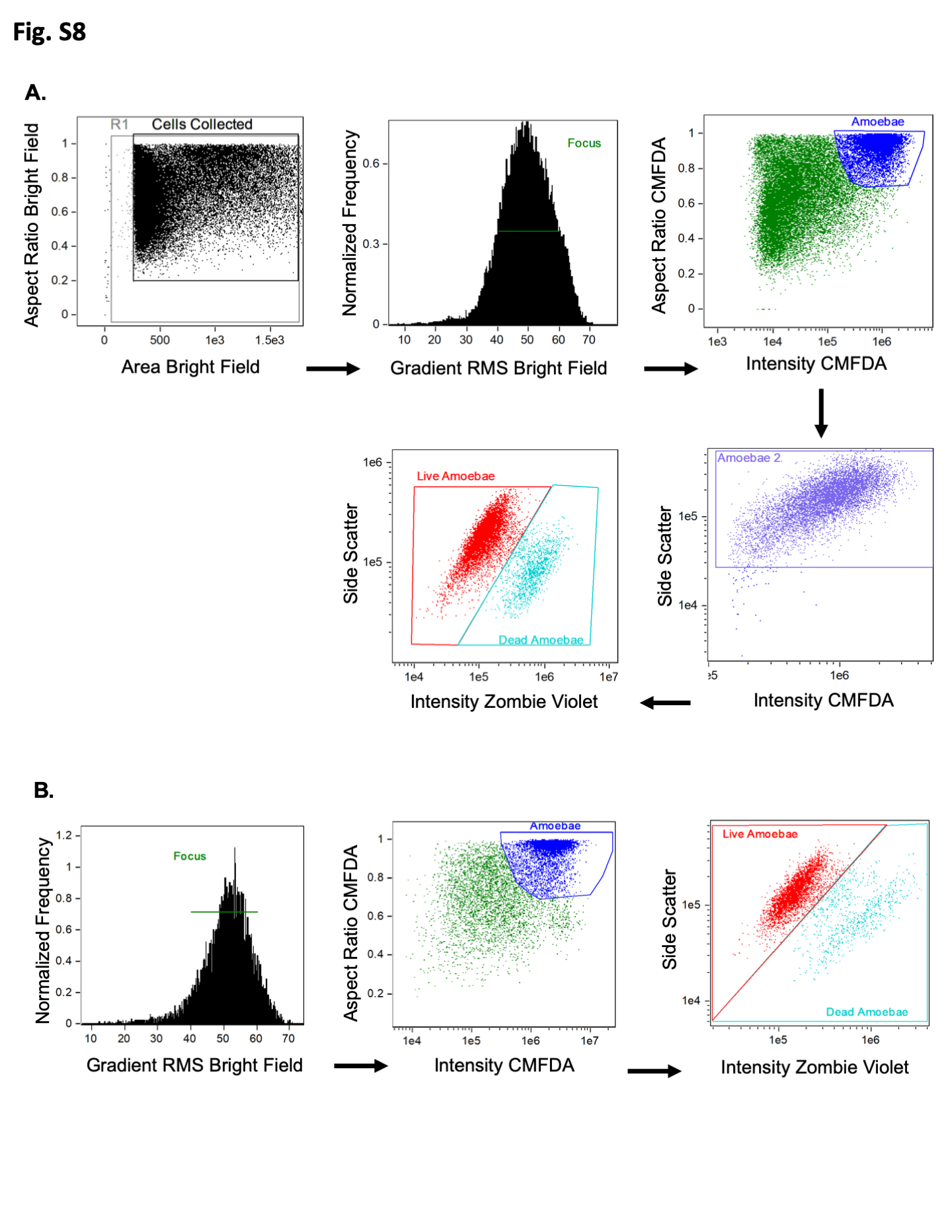

Supplement: FIG S8 [file mbio.03163-21-sf008.tif]

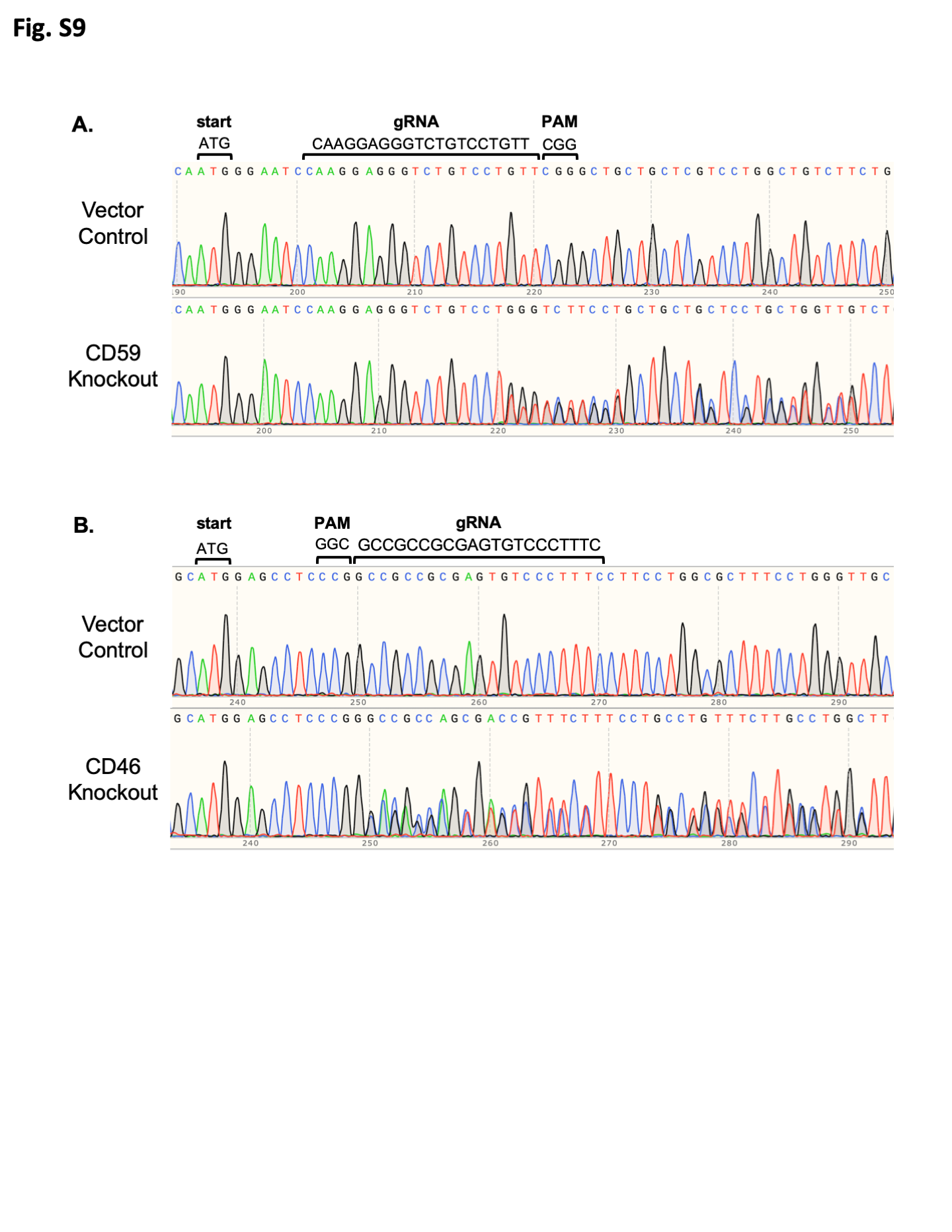

Supplement: FIG S9 [file mbio.03163-21-sf009.tif]
